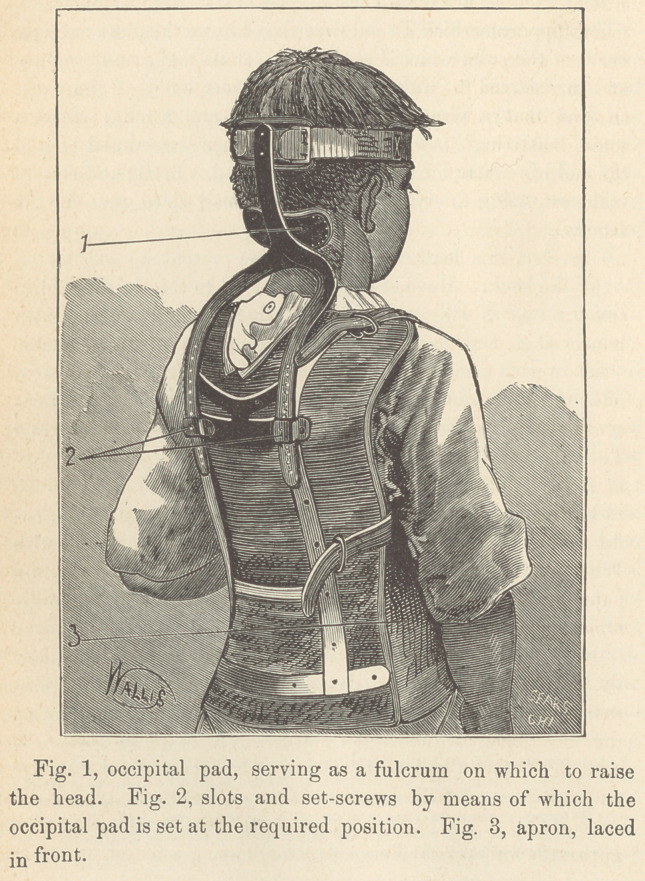# A New Apparatus for Pott’s Disease of the Cervical Vertebræ

**Published:** 1878-04

**Authors:** Wallace Blanchard

**Affiliations:** Chicago


### A NEW APPARATUS FOR POTT'S DISEASE OF THE CERVICAL VERTEBRÆ.

By Wallace Blanchard, M. D., Chicago.

The inadequacy, inconvenience and unsightliness of the instruments constructed for Pott's disease of the cervical vertebræ, have led me to devise an apparatus that for efficiency has far exceeded my expectations.

In this disease the carious body of the bone gives way, and the transverse spinous processes, from their posterior position, tip the head forward, till, as the disease advances, the chin may rest upon the chest. Any force that carries the head backward causes the transverse processes, which are seldom diseased, to serve as the fulcra for removing the pressure from the diseased body of the vertebræ; the density of their structure rendering them equal to the support of any reasonable weight.

While Dr. Sayre teaches this principle, he neglects its application in his apparatus for cervical Pott's disease, which he calls the "jury-mast;" the only act accomplished by it being the lifting directly upward of the head. It is uncleanly and disagreeable to wear, consisting as it does of "four or five layers" of plaster of Paris and crinoline, and a "mast," with a swing for the head, which attracts attention nearly as far as its wearer can be seen; matters of no small moment when peace of mind and out-door exercise are often considered a part of the treatment.

The apparatus which I shall describe obviates the disadvantages named, if the rules which I shall give for its make and application are observed.* Take a plaster-of-Paris mold of the back, and from that a plaster cast, quite thick and strong. Soak a piece of "skirting" (a form of saddler's leather dressed with oil and very stiff) in water for a day, and then bind it firmly and closely to the cast, using thirty to forty feet of small sized rope for the purpose.

When dry, this leather must of course present an exact contour of the back. Have steel strips riveted to this leather splint in such a way as will support it best, line the inner surface with chamois skin, and to each side sew an apron of light, pliable leather in such a manner that it may be laced over the abdomen.

Support a pad for the occiput on an arch formed by the joining of two steel strips that pass down over the back splint, and are there secured in such a manner as to be raised or lowered to any desired position, and there held by set-screws. Upward and backward from the occipital pad for three or four inches, extend a single strip of steel supporting two buckles, supplied with a band to pass around the head. Place the splint in its position on the back, with a light linen or cotton shirt next to the body, and lace the apron over the abdomen as tightly as can be borne with comfort by the patient. Add straps over each shoulder, only tight enough to steady the splint, and be sure that the splint extends high enough over the shoulders so that there shall be no

---

* I have been somewhat explicit in this article for the reason that it is too much the habit of certain gentlemen in large cities (not particularly in Chicago) to publish a treatment or describe an apparatus full enough to show its usefulness, and to just stop short of those details that would enable the average reader to make any practical use of the information given. The result is obvious. The doctors "in the provinces" send in their cases. I fail to see the practical difference between this and taking out a patent in the (ir)regular way.

downward pressure. The accuracy with which it fits every inequality of the back makes it quite immovable.

The occipital pad should now be in such a position that considerable force shall be required in lifting and carrying backward the head so as to place the occiput on the pad with the chin somewhat elevated; then buckle the strap over the forehead and the apparatus is applied as represented in the cut.

Fig. 1, occipital pad, serving as a fulcrum on which to raise the head. Fig. 2, slots and set-screws by means of which the occipital pad is set at the required position. Fig. 3, apron, laced in front.

The head is now firmly supported, and in such a manner that if there should be any excess of weight not carried by the instru ment, it is taken *entirely by the transverse processes.*

I first applied this apparatus to a girl of five years, from the southern part of the State. On my first visit I found her sitting on the bed, supporting her head with her hands, and was told that this had been for several weeks her almost constant position. There was considerable projection of the spine of the fourth cervical vertebra; she was pale and emaciated; exhibited nervous symptoms, and complained constantly of pain in the neck and throughout the thorax. On the application of the apparatus she was almost immediately relieved from pain. A mild nutritive tonic treatment was added, and she progressed so rapidly that at the end of a month I felt safe in letting her return home. A little over a year after, word was conveyed to me that the family physician considered her cured, and had discontinued the use of the instrument.

My second case, a boy of seven years, from Iowa, with symptoms and deformity not so marked, under the use of the apparatus, improved, and recovered in quite the same manner as the first.

The third, a boy of ten years, from Minnesota, was a case confided to the care of Dr. E. Bert, of this city, who called me in consultation with the intention of giving my apparatus a trial. The boy had a slight projection of the spines of the last cervical and first dorsal vertebræ, with considerable pain and nervous asthenia. A month after the apparatus was applied, he had so far improved that we considered it safe to let him return home. With his coat and cap on, this boy passed along the street without attracting the least attention to the fact that he was wearing a mechanical appliance.

In rotary-lateral curvatures, and advanced Pott's disease, below the level of the axillæ, Dr. Bryan's plaster-jacket applied during suspension, according to the method of Dr. Sayre, is undoubtedly the most efficient treatment yet devised. But in Pott's disease, in the lower half of the column, when the curvature is direct and not very marked, I prefer, after extension, to apply a steel supported leather back splint, as described, for the base of the apparatus for cervical Pott's disease, cutting a fenestrum of size sufficient to obviate pressure at the point of disease, and padding the shoulder straps which are to be buckled tightly over the shoulders. The patient can in this way be held in a nearly or quite perfect position, with immobility secured, and all the weight transferred from the bodies of the diseased vertebræ to their transverse processes.

I have found this treatment very successful in appropriate cases. The back splint used in this manner, originated, I believe, with the late Dr. J. S. Sherman, of this city, though Dr. Adams, of London, described a somewhat similar support at about the same time that this was introduced. The idea of wearing the immovable plaster-jacket for from two to four months at a time, as advocated by Dr. Sayre, is naturally repugnant to persons of ordinary cleanliness, and is to be avoided when any substitute that is just as effectual can be found.

Dr. Edmund Andrews tells me that his chief concern while using the jury-mast apparatus on young patients, has been their liability to slip the straps that pass under the jaw, so as to produce strangulation.

Mr. Chas. Degenhardt, surgical instrument maker, has done the steel work on my Pott's disease apparatus in a very creditable manner, but in a country town any skilled mechanic could do the work properly, if not so neatly, under the supervision of a surgeon having in view the results to be obtained.

More general attention should certainly be directed to this form of disease, considering its frequency, the large percentage of mortality, and the unsightly deformations so often left to the survivors.

## Figures and Tables

**Fig. 1 Fig. 2 Fig. 3 f1:**